# Development and validation of a prediction model for bronchopulmonary dysplasia using respiratory severity score

**DOI:** 10.1038/s41390-025-03862-z

**Published:** 2025-02-03

**Authors:** Takahiro Kanzawa, Fumie Kinoshita, Fumihiko Namba, Taihei Tanaka, Makoto Oshiro, Takahiro Sugiura, Yuichi Kato, Masafumi Miyata, Yasumasa Yamada, Osuke Iwata, Masahiro Hayakawa, Yoshiaki Sato, BPD Prediction Model Collaborative Clinical Research Team, Tetsuo Hattori, Hiroko Boda, Masayuki Fujino, Yuri Kawai, Arisa Kojima, Masahiko Manabe, Chiharuko Nakauchi, Yusuke Funato, Shigemitsu Kamino, Kennosuke Tsuda, Shin Kato, Kanji Muratmatsu, Mitsuhiro Haga, Asami Konishi, Haruka Noda, Osamu Shinohara, Seiji Hayashi, Yuko Murai, Kuniko Ieda, Kazuya Honbe, Masami Asai, Rika Nagasaki, Hikaru Yamamoto, Midori Yamada, Koji Takemoto, Yoshiaki Nagaya, Kazuyuki Yamamoto, Kazushi Yasuda, Satoru Kawai, Takehiko Yokoyama, Sayako Hamasaki, Naozumi Fujishiro, Ryo Tanaka, the Neonatal Research Network of Japan

**Affiliations:** 1 Department of Pediatrics, Nagoya University Graduate School of Medicine, Nagoya, Japan; 2 Division of Neonatology, Center for Maternal-Neonatal Care, Nagoya University Hospital, Nagoya, Japan; 3 Statistical Analysis Section, Department of Advanced Medicine, Nagoya University Hospital, Nagoya, Japan; 4 Department of Pediatrics, Saitama Medical Center, Saitama Medical University, Kawagoe, Saitama Japan; 5 Department of Neonatology, Japanese Red Cross Aichi Medical Center Nagoya Daini Hospital, Nagoya, Japan; 6 Department of Pediatrics, Japanese Red Cross Aichi Medical Center Nagoya Daiichi Hospital, Nagoya, Japan; 7 Department of Pediatrics and Neonatology, Toyohashi Municipal Hospital, Toyohashi, Aichi Japan; 8 Department of Pediatrics, Anjo Kosei hospital, Anjo, Aichi Japan; 9 Department of Pediatrics, Fujita Health University School of Medicine, Toyoake, Aichi Japan; 10 Department of Perinatal and Neonatal Medicine, Aichi Medical University, Nagakute, Aichi Japan; 11 Department of Pediatrics and Neonatology, Nagoya City University Graduate School of Medical Sciences, Nagoya, Japan; 12 Medical Corporation Kishokai, Nagoya, Japan; 13 Department of Pediatrics, Handa City Hospital, Handa, Aichi Japan; 14 Department of Pediatrics, Okazaki City Hospital, Okazaki, Aichi Japan; 15 Department of Pediatrics, Komaki City Hospital, Komaki, Aichi Japan; 16 Department of Pediatrics, Tosei General Hospital, Seto, Aichi Japan; 17 Department of Pediatrics, Kojunkai Social Medical Corporation Daido Hospital, Nagoya, Japan; 18 Department of Pediatrics, Kainan Hospital Aichi Prefectural Welfare Federation of Agricultural Cooperatives, Yatomi, Aichi Japan; 19 Department of Neonatology, Toyota Memorial Hospital, Toyota, Aichi Japan; 20 Department of Pediatrics, Kariya Toyota General Hospital, Kariya, Aichi Japan; 21 Department of Pediatrics, Konan Kosei Hospital, Konan, Aichi Japan; 22 Department of Pediatrics, Ichinomiya municipal hospital, Ichinomiya, Aichi Japan; 23 Department of Pediatrics, Nagoya City University West Medical Center, Nagoya, Japan; 24 Department of Pediatric Cardiology, Aichi Children’s Health and Medical Center, Obu, Aichi Japan; 25 Department of Neonatology, Aichi Children’s Health and Medical Center, Obu, Aichi Japan

## Abstract

**Background:**

To develop and validate a prediction model for severe bronchopulmonary dysplasia (BPD) that integrates the respiratory severity (RS) score with early postnatal risk factors.

**Methods:**

This retrospective cohort study included preterm infants born at less than 32 weeks gestation or with a birth weight of less than 1500 g, from Aichi Prefecture (training dataset) and Saitama Medical University (validation dataset) from April 1, 2016, to March 31, 2020. The primary outcome was severe BPD, defined as the use of home oxygen therapy or death due to BPD. We used classification and regression tree (CART) analysis to explore the relationship between outcomes and BPD risk factors in the training dataset.

**Results:**

The incidence of severe BPD was 149 out of 2026 (7.3%) in the training dataset and 35 out of 387 (8.9%) in the validation dataset. CART analysis identified gestational age and the RS score as significant predictors of outcome in the day 7 and day 14 models, with C-statistics of 0.789 and 0.779, respectively. When applied to the validation dataset, these models achieved C-statistics of 0.753 and 0.827, respectively.

**Conclusion:**

Our prediction models demonstrated the ability to predict severe BPD, with the RS score being a crucial predictor.

**Impact:**

- Many existing prediction models for bronchopulmonary dysplasia (BPD) use multiple predictors, and do not provide specific cutoff values, which complicates their clinical application.
- To address this issue, we developed a prediction model for severe BPD based on a score derived from mean airway pressure and inhaled oxygen concentration at 1–2 weeks of age.
- This user-friendly model can be easily integrated into clinical practice, facilitating treatment decisions based on predicted probabilities.

## Introduction

Approximately 50% of very preterm infants born between 22 and 27 weeks of gestational age (GA) have bronchopulmonary dysplasia (BPD) at 36 weeks corrected GA, and the incidence of BPD has remained high for the past 30 years.^1^ Reducing the incidence of BPD is a critical issue, as it increases adverse neurodevelopmental outcomes in infancy,^2^ rehospitalization, and pulmonary hypertension.

Some examples of strategies for preventing the development or progression of BPD may include pharmacotherapy, such as caffeine;^3^ and corticosteroids^4,5^ administration of noninvasive surfactants;^6^ Nasal high flow therapy^7^ and neurally adjusted ventilatory assist ventilation.^8^ However, because therapeutic agents for BPD can cause side effects at the same time, avoiding unnecessary treatment for populations at lower risk of BPD and selectively treating only those with severe BPD may be beneficial in improving the outcome of preterm infants. Furthermore, because novel therapies such as stem cell therapy for BPD^9^ are expensive, it is necessary to select infants who require the treatment based on health and economic factors. However, it is difficult to predict the onset and severity of BPD early in life. It is therefore critical to develop a “BPD prediction model” to predict severe BPD in the early stages of life.

Several previous studies have developed BPD prediction models using various early potential risk factors.^10–12^ These systematic reviews reported BPD prediction models using clinical parameters universally available from immediately after birth to 2 weeks of age. For example, the timing of obtaining the predictive factors differs depending on the report, such as from immediately after to 2 weeks after birth, whereas the common factors include the GA at birth, birth weight (BW), sex, multiple pregnancies, Apgar score, chronic maternal hypertension, need for resuscitation at birth, respiratory distress syndrome (RDS), patent ductus arteriosus (PDA), and intraventricular hemorrhage (IVH). However, these models are challenging to apply in clinical practice due to their use of numerous predictors and the absence of specific cutoff values for each predictor, which complicates their practical implementation. Furthermore, their C-statistics ranged from 0.50 to 0.76, after external validation.^11^ It is thus critical to use predictors with high predictive accuracy, to create simple models with fewer predictors, and to define their cutoff values. As a candidate, we focused on the respiratory severity (RS) score, which is calculated by multiplying mean airway pressure (MAP) and fraction of inspiratory oxygen (FiO_2_) during ventilation. The RS score has also been shown to predict the development of severe BPD, death, duration of ventilation, and pulmonary hypertension.^13–15^ Using a cutoff value of 3.0 for predicting severe BPD, the positive and negative predictive values at 14 days of age were 79.07% and 61.54% respectively. However, the cutoff values for the RS score used in these prediction models are based on single-center data, and the prediction models have not been externally validated. Furthermore, the cutoff values can differ depending on the patient population, treatment outcome, and other variables. Therefore, it is ideal to develop models based on multi-center data and then validate them externally.

This study aimed to develop a BPD prediction model that could be more simply used in clinical practice by combining the RS score with clinical parameters that can be obtained early after birth. Furthermore, a population-based study was conducted to establish higher-quality cut-off values for the predictive factors, and classification and regression tree (CART) analysis was performed.

## Method

### Subjects

This was a retrospective cohort study that included all 21 perinatal and neonatal centers in Aichi Prefecture, as well as the Department of Pediatrics, Saitama Medical Center, and Saitama Medical University in Saitama Prefecture. The oxygen saturation target was 85–95%, and no difference was observed between the two cohorts. The population of Aichi Prefecture is 6 million, with approximately 50,000 births per year. We included preterm infants born at the above centers between April 1, 2016 and March 31, 2020, at less than 32 weeks gestation or less than 1500 g BW, and who received invasive or noninvasive ventilation for at least seven days. To ensure the simple use in clinical practice, the development of a prediction model, including up to five or more prediction factors, was considered in advance. To ensure the number of events per variable is ten or more,^16^ at least 50 patients with event occurrence are required. The Neonatal Research Network database in Japan indicated that the incidence of BPD requiring home oxygen therapy (HOT) is approximately 8%,^17^ with the target period calculated based on the annual number of births of eligible cases in Aichi Prefecture. Exclusion criteria included neonatal deaths within 7 days of birth, neonatal transport from the birth institution to another healthcare facility between 7 and 28 days of age, cases with major malformations affecting respiratory and circulation, and chromosomal abnormalities.

### Maternal and neonatal variables

Each term was defined using the definitions from the Neonatal Research Network database Japan.^18^ Severe BPD was defined as the use of HOT, home ventilation upon discharge, or death from BPD during hospitalization. Clinical chorioamnionitis (CAM) was defined using the diagnostic criteria established by Lencki et al.^19^ Histological CAM was defined as Stage 1 or higher on the Blanc classification.^20^ Symptomatic patent ductus arteriosus (PDA) was defined as any circulatory condition that required treatment. Intraventricular hemorrhage (IVH) was defined as any grade of classification by Volpe et al.^21^ Sepsis was defined as the presence of bacterial or fungal pathogens in a blood culture. The values of FiO_2_ and MAP were extracted from the respiratory settings in the medical records. The recording interval differed between facilities but was generally every 1 to 3 h, and the most frequent values during the 24 h of the subject days (7 and 14 days old) were chosen as representative values. The RS score was calculated using the following formula, RS score = FiO_2_ × MAP. If MAP was not recorded in the medical record, it was calculated using the following formula from the values of maximum airway pressure (PIP), positive end-expiratory pressure (PEEP), ventilation frequency (RR), and inspiratory time (Ti), which were adopted according to the same definition: MAP = PEEP + (PIP - PEEP× Ti × RR / 60).^22^

### Statistical analysis

Data obtained from the perinatal and neonatal centers in Aichi Prefecture was used as the training dataset, while data collected from the Department of Pediatrics, Saitama Medical Center, Saitama Medical University was used as the validation dataset. In each dataset, the χ2 test was used for categorical variables and the Mann–Whitney’s U test for continuous variables to compare the demographic and clinical characteristics of infants with and without severe BPD or in the training and validation datasets. The analysis was performed using JMP pro 16.1.0 (SAS Institute Inc., Cary, NC) and SAS version 9.4 (SAS Institute, Inc., Cary, NC). Among the factors identified as BPD risk factors in previous reports^10–12,23^ and available from the Neonatal Research Network database Japan, the following BPD risk factors were chosen as pre-determined covariate candidates, GA, BW, BW standard deviation score (SDS), body length at birth, body length at birth SDS, sex, clinical CAM, histologic CAM, premature rupture of membrane (PROM), maternal steroid, Apgar score at 1 min and 5 min, respiratory distress syndrome (RDS), pulmonary hemorrhage, symptomatic PDA, IVH, sepsis, intubation at birth, RS score (7 and 14 days of age).

Predictive models with vs. without severe BPD were developed using CART analysis. CART analysis uses machine learning to identify explanatory variables and cutoff values that influence the objective variable.^24–26^ The cutoff value of the root node or daughter node is automatically selected using the likelihood-ratio χ2 statistic. The total number of nodes was limited to 4 to create simpler models with fewer predictors. Sensitivity analysis was performed to create a decision tree by cost-complexity pruning with tenfold cross-validation. The covariates to be adapted to the model were chosen from a list of pre-determined candidate covariates, taking into account the *p*-values from the univariate analysis. If there was a strong correlation (correlation coefficient >0.90) between selected covariates, factors commonly used in routine practice were added as covariates and adjusted so that the variance inflation factor (VIF) was all less than 5. A case with a missing value for an adapted covariate was excluded from the training dataset for the CART analysis. The predictive performance of the prediction model was evaluated using the C-statistic. Then, the predictive model was applied to the validation dataset. At this time, the generalizability of the prediction model, in addition to the total validation dataset, confirmed the application of the prediction model to a subgroup consisting of infants at <28 weeks of gestation and infants treated with maternal steroids, and the true probability for each node was computed. Finally, a calibration plot was created using the predicted and actual probabilities for each value of the final node of the decision tree.

This study was approved by the Institutional Ethics Committee of Nagoya University (approval number: 2020-0386).

## Result

### Training dataset

**Fig. 1:**
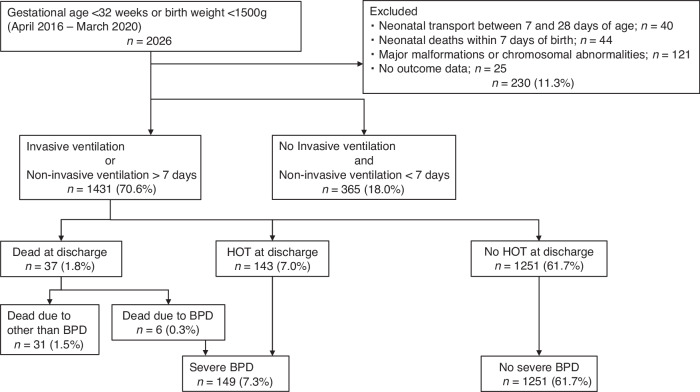
Flowchart of the study population used in the training dataset.

Figure 1 depicts a flowchart for this study. There were 2026 preterm infants with a GA of less than 32 weeks, or a BW of less than 1500 g in the training cohort. The rates of eligible and severe BPD were 1431 (70.6%) and 149 (7.3%), respectively. Table 1 shows the demographic characteristics of the study population. The cause of death is shown in Supplemental 1. When comparing the groups with and without severe BPD, median RS scores (interquartile range) were 1.89 (1.31–2.65) and 1.26 (0.84–1.45) at 7 days of age, and 2.20 (1.43–3.81) and 1.05 (0–1.43) at 14 days of age respectively, which were significantly higher in the group with severe BPD at both days of age. Furthermore, several factors, including GA, BW, body length at birth, clinical CAM, histologic CAM, PROM, Apgar scores at 1 min and 5 min, RDS, symptomatic PDA, IVH, sepsis, intubation at birth, and RS score, differed significantly different between those with and without severe BPD.

**Table 1:** Demographic and clinical characteristics of the study population used in the training dataset.

	Severe BPD (*n* = 149)	No severe BPD (*n* = 1251)	*p*-value
Gestational age (week)	25.4 (23.7–27.6)	29.4 (27.2–31.0)	<0.0001
Birth weight (g)	712 (545–925)	1148 (861–1372)	<0.0001
Birth weight (SDS)	−0.5 ( − 1.2–0.3)	−0.64 ( − 1.7–0.1)	0.0779
Body length at birth (cm)	31.3 (28.7–34.7)	37.0 (33.8–39.5)	<0.0001
Body length at birth (SDS)	−0.43 ( − 1.3–0.2)	−0.49 ( − 1.4–0.3)	0.9707
Male (*n*,%)	77 (51.7)	625 (50.0)	0.6918
Clinical CAM (*n*,%)	41 (27.7)	145 (11.8)	<0.0001
Histologic CAM (*n*,%)	67 (47.2)	308 (25.9)	<0.0001
PROM (*n*,%)	57 (38.8)	338 (28.5)	0.0099
Maternal steroid (*n*, %)	90 (63.8)	771 (64.0)	0.9614
1 min Apgar score	3 (1–5)	5 (3–7)	<0.0001
5 min Apgar score	6 (4–8)	8 (6–9)	<0.0001
RDS (*n*,%)	111 (74.5)	741 (59.4)	0.0003
Pulmonary hemorrhage (*n*,%)	4 (2.68)	14 (1.13)	0.1116
Symptomatic PDA (*n*,%)	73 (49.3)	433 (35.6)	0.0011
IVH (*n*,%)	33 (22.5)	147 (12.1)	0.0005
Sepsis (*n*,%)	22 (14.97)	60 (4.94)	<0.0001
Intubation at birth (*n*,%)	130 (87.2)	880 (71.2)	<0.0001
RS score (day7)	1.89 (1.31 − 2.65)	1.26 (0.84–1.45)	<0.0001
RS score (day14)	2.20 (1.43 − 3.81)	1.05 (0.0–1.43)	<0.0001
Inhaled corticosteroid (*n*,%)	72 (48.3)	213 (17.7)	<0.0001
Systemic corticosteroid (*n*,%)	79 (55.6)	154 (19.47)	<0.0001
HFO (*n*,%)	88 (64.2)	257 (25.8)	<0.0001
Length of Invasive ventilation (day)	43 (4–68)	2 (1–12)	<0.0001
Length of Noninvasive ventilation (day)	39 (16 − 59)	27 (10–41)	<0.0001
Age at discharge (day)	137 (104–168)	73 (54–100)	<0.0001

Values are presented as median (first quartile to third quartile) or numbers (%).

Severe BPD is defined as the use of home oxygen therapy (HOT) or home ventilation at discharge or death from BPD during hospitalization.

*BPD* bronchopulmonary dysplasia, *SDS* standard deviation score, *CAM* chorioamnionitis, *PROM* premature rupture of membrane, *RDS* respiratory distress syndrome, *PDA* patent ductus arteriosus, *IVH* intraventricular hemorrhage, *RS* score respiratory severity score, *HFO* high-frequency oscillation.

### Prediction model

Of the pre-determined candidate covariates, the following risk factors showed significant differences between groups with and without severe BPD: GA, BW, body length at birth, clinical CAM, histologic CAM, PROM, Apgar scores at 1 min and 5 min, RDS, symptomatic PDA, IVH, sepsis, intubation at birth, and the RS score. Due to a strong correlation (*r* > 0.90) between body length at birth and BW among the candidate covariates, BW—commonly used in routine practice—was adopted as a covariate, leading to exclusion of body length at birth. With body length at birth excluded, all VIFs were less than 5. Therefore, the following covariates were used to develop the prediction model: RS score, GA, BW, clinical CAM, histologic CAM, PROM, Apgar scores at 1 min and 5 min, RDS, symptomatic PDA, IVH, sepsis, and intubation at birth.

**Fig. 2:**
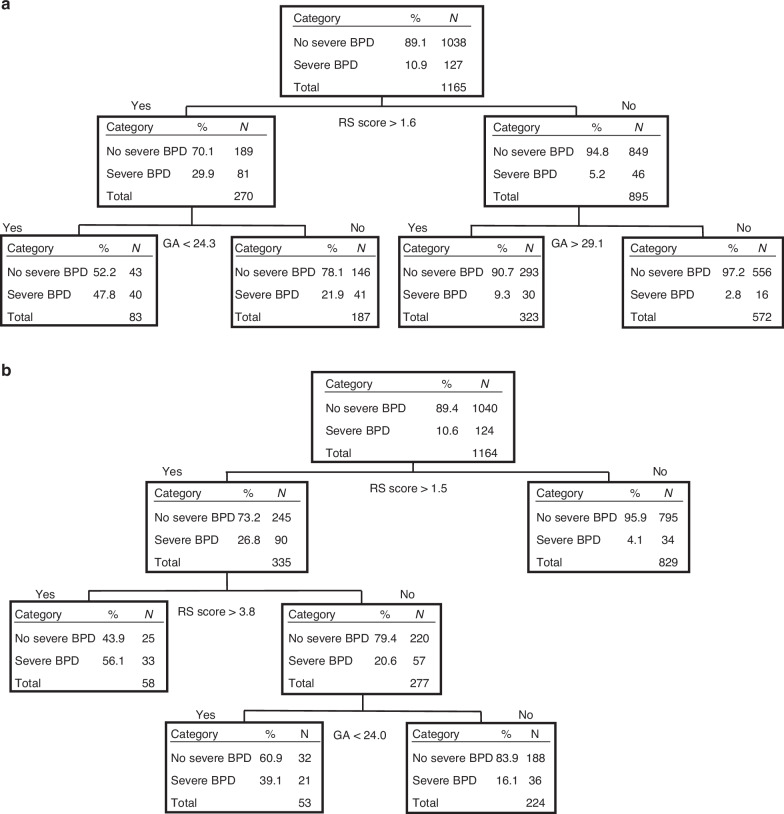
Classification and regression tree (CART) model for predicting severe BPD at 7 and 14 days of age. **a** Postnatal day 7 model. **b** Postnatal day 14 model. Predictive models with and without severe BPD were created using classification and regression tree (CART) analysis. The cutoff value for each root node or daughter node is automatically selected according to the likelihood-ratio chi-square statistic. The following covariates were used to create the prediction model, RS score, gestational age, birth weight, clinical CAM, histologic CAM, PROM, 1 min Apgar score, 5 min Apgar score, RDS, symptomatic PDA, IVH, Sepsis, and intubation at birth. The percentage represents predicted probabilities, while the *N* value represents the number of infants in each category. BPD is bronchopulmonary dysplasia, CAM is chorioamnionitis, PROM is premature rupture of membrane, RDS is respiratory distress syndrome, PDA is patent ductus arteriosus, IVH is intraventricular hemorrhage, and RS score is respiratory severity score.

**Fig. 3:**
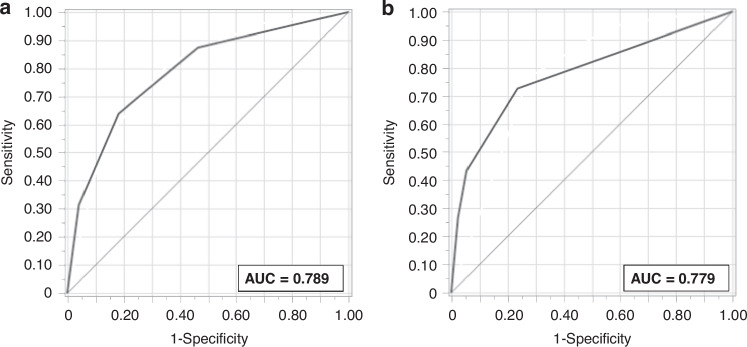
Receiver operating characteristic (ROC) curves for the CART model for predicting severe BPD at 7 and 14 days of age (Training dataset). **a** Postnatal day 7 model. **b** Postnatal day 14 model.

Using CART analysis, two outcome prediction models were developed for different days of age: the “Postnatal Day 7 Model,” and the “Postnatal Day 14 Model.” Since the total number of nodes was limited to four to create simpler models with fewer predictors, two of the 13 covariates included in the CART model were adopted as predictors: the RS score and GA. Although cases with missing data for the adapted covariates were excluded from the CART analysis dataset, no significant differences in clinical characteristics were observed between the included and excluded cases (Supplemental 2). The Postnatal day 7 (Figs. 2a and 3a) and day 14 (Figs. 2b and 3b) models were adjusted for RS score and GA, with C-statistics of 0.789 and 0.779 respectively. Sensitivity analysis was performed to create a decision tree by cost-complexity pruning with tenfold cross-validation. The number of nodes in decision trees for days 7 and 14 were both eight, with c-statistics of 0.751 and 0.778 for each model, respectively. Since the difference in c-statistics was not observed, a model with the number of nodes limited to four was adopted as the prediction model, with careful consideration of its clinical usefulness.

### External validation

**Fig. 4:**
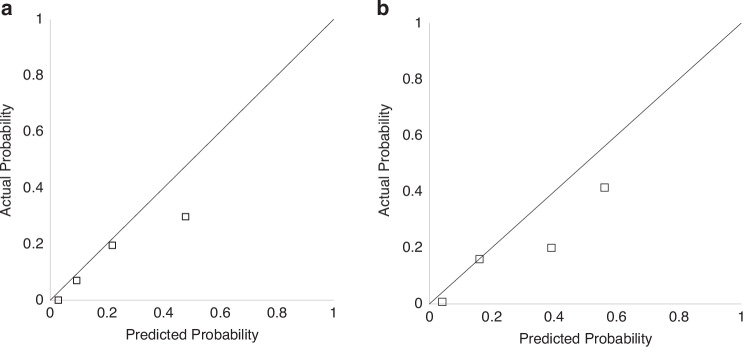
Calibration plot using validation dataset. **a** Postnatal day 7 model. **b** Postnatal day 14 model.

The validation cohort included 387 preterm infants with a GA of less than 32 weeks, or a BW of less than 1500 g in the validation cohort. The rates of eligible and severe BPD were 284 (77.3%) and 35 (8.9%), respectively (Supplemental 3). The validation dataset included infants with earlier GA than the training dataset, with median (interquartile range) weeks of gestation of 27.7 (25.4–29.1) and 29.2 (26.8–30.9) weeks, respectively. Furthermore, histological CAM and systemic corticosteroid use were more common in the validation dataset (Table 2). When the validation dataset was applied to Postnatal day 7 and 14 models, the C-statistics were 0.753 and 0.827, respectively (Supplemental 4). Moreover, when these models were used for infants born before 28 weeks of gestation and those treated with maternal steroids, the C-statistics measured were 0.579 and 0.692 for the subgroup of infants under 28 weeks and 0.777 and 0.873 for the maternal steroid subgroups, respectively (Supplemental 5). Figure 4 shows calibration plots. “Postnatal day 7 model” and “Postnatal day 14 model” tended to overestimate when these predicted probabilities were high, yet they still fit reasonably well. The calibration plot created using the subgroup consisting of infants at <28 weeks of gestation and those treated with maternal steroids was also reasonably fit (Supplemental 6).

**Table 2:** Comparison of demographic and clinical characteristics of the study populations used in the training and validation datasets.

	Training dataset (*n* = 1400)	Validation dataset (*n* = 277)	*p*-value
Gestational age (week)	29.2 (26.8–30.9)	27.7 (25.4–29.1)	<0.0001
Birth weight (g)	1096 (806–1358)	926 (672–1161)	<0.0001
Birth weight (SDS)	−0.6 ( − 1.6–0.1)	−0.6 ( − 1.5–0.4)	0.1524
Body length at birth (cm)	36.5 (33.0–39.0)	34.1 (31.0–37.0)	<0.0001
Body length at birth (SDS)	−0.47 ( − 1.4–0.3)	−0.51 ( − 1.3–0.12)	0.3341
Male (*n*,%)	702 (50.1)	153 (55.2)	0.1214
Clinical CAM (*n*,%)	186 (13.5)	36 (13.0)	0.8232
Histologic CAM (*n*,%)	375 (28.2)	129 (47.4)	<0.0001
PROM (*n*,%)	395 (29.6)	84 (30.3)	0.8128
Maternal steroid (*n*, %)	861 (64.0)	172 (62.0)	0.5449
1 min Apgar score	5 (3–7)	5 (4–6)	0.3112
5 min Apgar score	7 (6–9)	7 (6–8)	0.0171
RDS (*n*,%)	852 (61.0)	200 (72.2)	0.0004
Pulmonary hemorrhage (*n*,%)	18 (1.29)	5 (1.82)	0.4952
Symptomatic PDA (*n*,%)	506 (37.0)	121 (44.3)	0.0239
IVH (*n*,%)	180 (13.2)	43 (15.5)	0.3072
Sepsis (*n*,%)	82 (6.0)	21 (7.6)	0.3216
Intubation at birth (*n*,%)	1010 (72.9)	238 (85.9)	<0.0001
RS score (day7)	1.26 (1.00–1.55)	1.63 (1.05–2.2)	0.0004
RS score (day14)	1.19 (0.0–1.68)	1.84 (1.05–3.3)	<0.0001
Inhaled corticosteroid (*n*,%)	285 (21.0)	28 (12.3)	0.0023
Systemic corticosteroid (*n*,%)	233 (25.0)	136 (49.2)	<0.0001
HFO (*n*,%)	345 (30.4)	152 (60.8)	<0.0001
Length of Invasive ventilation (day)	3 (1–18)	17 (3–46)	<0.0001
Length of Noninvasive ventilation (day)	28 (10–43)	32 (18–43)	0.0309
Age at discharge (day)	77 (56–109)	103 (79–136)	<0.0001
Severe BPD (*n*,%)	149 (10.6)	35 (12.6)	0.3323

Values are presented as median (first quartile to third quartile) or numbers (%).

*BPD* bronchopulmonary dysplasia, *SDS* standard deviation score, *CAM* chorioamnionitis, *PROM* premature rupture of membrane, *RDS* respiratory distress syndrome, *PDA* patent ductus arteriosus, *IVH* intraventricular hemorrhage, *RS* score respiratory severity score, *HFO* high-frequency oscillation.

## Discussion

In this study, we have developed a method for predicting severe BPD that can be easily applied in clinical practice using GA and RS scores in the first 1–2 weeks of life. We developed a prediction model through a retrospective population-based study in Aichi Prefecture, and its predictive accuracy was consistent across external validation using datasets from another region.

Furthermore, the predictive ability was maintained even when this predictive model was used to subgroups considered to be at higher risk; therefore, the generalizability was guaranteed. We developed a BPD prediction model using only two predictors, applicable as early as day 7 or 14 of age, which demonstrated high predictive accuracy with C-statistics of 0.789 and 0.779, respectively. This model was based on a population-based study encompassing all eligible cases from all NICUs in one prefecture, including 1400 cases with nearly 150 events. Additionally, the model underwent external validation and calibration testing. Onland et al. examined 26 BPD prediction models and found that when external validity was applied to 19 prediction models with covariates consistent with the authors’ data set, the C-statistics ranged from 0.50 to 0.76.^11^ In these prediction models, only four used external validation, and none included calibration. Romijn et al. conducted a systematic review of prediction models for death or BPD at 14 days of age or younger.^12^ In this, the median c-statistic during model development was reported as 0.84 (range 0.43–1.00), while the median C-statistic during external validation was 0.77 (range 0.41–0.97). However, most prediction models are considered at high risk of bias due to small sample size (events per variable <10) or insufficient handling of missing data. Furthermore, these models used more predictors: the number of predictors in the final model was 6 (range 2–21). The model, which predicts with high accuracy using fewer predictors is ideal because it makes the model easier to use in clinical practice. Overall, the model in this study was designed and validated appropriately, has less bias than previously reported models, and is more useful and user-friendly in clinical practice.

In this study, we focused on the RS score as an indicator of respiratory status. The Oxygenation Index (OI) is an effective indicator of poor oxygenation in ventilated infants;^27^ however, arterial blood samples are required to determine PaO_2_. Therefore, to avoid the invasiveness of blood sampling, the RS score was proposed as a noninvasive indicator for assessing the state of poor oxygenation. There was a strong correlation (R^2^ = 0.982) between RS score and OI when oxygen saturation (SpO_2_) was kept between 88 and 94%.^28^ This condition would apply to the respiratory management of preterm infants with SpO_2_ targets of less than 95%.^29^ There have also been several reports that show an association between RS score and complications in preterm infants. Malkar et al. evaluated RS scores in very low BW infants who required ventilation until the 30th day of life and found that an RS score >6.0 on the 30th day was associated with higher mortality and a longer duration of ventilation.^14^ Jung et al. also conducted a retrospective study on RS scores in preterm infants born at less than 28 weeks’ gestation who were ventilated for more than one week at a single center. They reported that the sensitivity and specificity for predicting death or severe BPD were 57.6% and 81.63%, respectively, with an RS score cutoff value of 3.0 at 14 days of age.^13^ It should be noted that our BPD prediction model, which simply combined GA and the RS score was able to predict death or severe BPD as early as 7 days of age, surpassing previous reports. Furthermore, both postnatal day 7 and 14 models maintained their predictive accuracy in external validation using datasets from various health regions.

In our prediction model, GA was included alongside the RS score as a predictor. Seo et al. found that GA, PROM, and weight-standardized RS scores were all significantly associated with BPD-induced pulmonary hypertension in preterm infants born at less than 30 weeks’ gestation.^15^ It implies that the combination of RS score and GA is critical for developing an effective prediction model.

Because some treatments for BPD have side effects, it is preferable to target only those with severe BPD rather than administering these treatments prophylactically to all infants. Therefore, developing BPD prediction models that can be used early after birth is crucial for improving outcomes in preterm infants. One example of a BPD treatment associated with adverse effects is corticosteroids. Several intervention trials have been conducted to evaluate their efficacy. A Cochrane review published in 2021 found that systemic corticosteroids administered before 7 days of age reduced the composite outcome of death or BPD at 36 weeks’ corrected age (RR 0.89, 95% CI 0.84 to 0.94); however, also increased the risk of cerebral palsy (RR 1.43, 95% CI 1.07 to 1.92) and gastrointestinal perforation (RR 1.84, 95% CI 1.36 to 2.49).^5^ Therefore, corticosteroid treatment for BPD is not recommended as a universal approach due to its associated side effects. Conversely, stratifying the target population -based on the risk of developing BPD has been suggested to improve treatment outcomes. A 2014 meta-analysis of 20 randomized clinical trials found that postnatal corticosteroids increased the risk of death or cerebral palsy in populations with less than a 33% risk of BPD, while they reduced the risk of these adverse events in populations with a 60% or greater risk of BPD.^30^ Therefore, accurately identifying high-risk groups and targeting therapeutic interventions accordingly can maximize treatment benefits. The model developed in this study can identify high-risk infants early and with high accuracy, allowing for targeted treatment and potentially improving outcomes for preterm infants.

The strength of this study is that we created this very simple BPD prediction model through a population-based study that included all eligible cases in all NICUs in one prefecture, totaling 1400 cases and nearly 150 events. This allowed the model to be free of facility selection bias and applicable to cases from a large number of facilities. This study includes Japanese infants, which few previous studies have investigated (mostly White population). Conversely, this study has several limitations. The study involved cases of GA between 28 to 32 weeks, during which severe BPD occurrences were minimal, and maternal steroid use was restricted. To assess these impacts, validation utilized a subgroup dataset comprising infants under 28 weeks of gestation and those administered maternal steroids. We generated a calibration plot that showed a strong correlation between the predicted and actual probabilities. These findings suggest good generalizability. Data on demographic and clinical characteristics were obtained from the Neonatal Research Network database in Japan, which did not have access to all maternal and drug administration information for infants, which could influence the onset and severity of BPD. The criteria for HOT and the definition of severe BPD may differ between institutions. However, the fact that the prediction accuracy was maintained even when a training dataset from a population-based study and a validation dataset from a different healthcare area were used suggests that the generalization performance as a prediction model is guaranteed. Future plans include establishing of a prospective registry to assess outcomes using the same criteria and validating the accuracy of the BPD prediction model.

Finally, we created a severe BPD prediction model that is easily applicable in clinical practice. Furthermore, its accuracy was maintained following external validation with datasets from various healthcare areas. This simple prediction model developed in this study is easily applicable in clinical practice, allowing for treatment selection based on predicted probability.

## Supplementary information

Supplemental Material

## Data Availability

All data are available upon request.
