# Supplementary material for: Development and validation of a prediction model for bronchopulmonary dysplasia using respiratory severity score
Source: Pediatr Res. 2025 Feb 3;98(2):577–84. doi: 10.1038/s41390-025-03862-z (PMC12454154; doi:10.1038/s41390-025-03862-z)
Supplement: Supplementary file 1 — Supplemental Material [file 41390_2025_3862_MOESM1_ESM.pdf]

Supplement 1. Death cause of the study population used in the training dataset

Causes of death	
Sepsis (n)	10
Necrotizing enterocolitis (n)	8
BPD (n)	6
Metabolic acidosis (n)	1
Apnea (n)	1
IVH (n)	1
Renal failure (n)	1
Unknown (n)	9
Total (n)	37

Values are presented as numbers.

BPD (bronchopulmonary dysplasia); IVH (intraventricular hemorrhage)

Supplement 2. Demographic and clinical characteristics of eligible and excluded cases.

	Postnatal day 7 model			Postnatal day 14 model		
	**Eligible cases (n = 1165)**	**Excluded cases (n = 235)**	**p-value**	**Eligible cases (n = 1164)**	**Excluded cases (n = 236)**	**p-value**
Gestational age (week)	29.1 (26.7–30.7)	29.7 (27.1–31.5)	0.0021	29.1 (26.7–30.7)	29.7 (27.1–31.5)	0.0022
Birth weight (g)	1079 (797–1341)	1188 (877–1415)	0.0037	1079 (796–1342)	1186 (884–1415)	0.0026
Birth weight (SDS)	−0.6 (−1.6–0.1)	−0.7 (−1.9–0.1)	0.2582	−0.6 (−1.6–0.1)	−0.7 (−1.9–0.1)	0.3398
Body length at birth (cm)	36.3 (33.0–39.0)	37.0 (34.0–40.0)	0.0050	36.3 (33.0–39.0)	37.1 (34.0–40.0)	0.0034
Body length at birth (SDS)	−0.4 (−1.4–0.3)	−0.7 (−1.3–0.2)	0.3500	−0.4 (−1.4–0.3)	−0.7 (−1.3–0.2)	0.3080
Male (n,%)	576 (49.4)	126 (53.6)	0.2429	572 (49.1)	130 (55.08)	0.0959
Clinical CAM(n,%)	151 (12.9)	35 (16.4)	0.1729	153 (13.1)	33 (15.4)	0.3704
Histologic CAM(n,%)	329 (28.2)	46 (28.2)	0.9959	327 (28.1)	48 (29.2)	0.7542
PROM(n,%)	348 (29.8)	47 (27.8)	0.5835	347 (29.8)	48 (28.2)	0.6742
Maternal steroid(n, %)	760 (65.7)	101 (53.4)	0.0011	758 (65.6)	103 (54.2)	0.0024
1min Apgar score	5 (3–7)	5 (3–8)	0.1062	5 (3–7)	5 (3–8)	0.0772
5min Apgar score	7 (6–9)	8 (6–9)	0.1047	7 (6–9)	8 (6–9)	0.0777
RDS (n,%)	717 (61.6)	135 (58.2)	0.3387	717 (61.6)	135 (57.9)	0.2961
Pulmonary hemorrhage (n,%)	15 (1.29)	3 (1.29)	1.0000	15 (1.29)	3 (1.29)	0.9934
Symptomatic PDA (n,%)	450 (38.6)	56 (27.9)	0.0035	449 (38.6)	57 (28.2)	0.0049
IVH (n,%)	142 (12.2)	38 (19.3)	0.0065	142 (12.2)	38 (19.2)	0.0072
Sepsis (n,%)	72 (6.2)	10 (5.1)	0.5468	72 (6.2)	10 (5.1)	0.5348
Intubation at birth (n,%)	859 (73.7)	151 (68.6)	0.1186	859 (73.8)	151 (68.3)	0.0933
RS score (day7)	1.26 (1.05–1.56)	1.26 (0.0–1.50)	0.2982	1.26 (1.05–1.56)	1.26 (0.0–1.58)	0.8393
RS score (day14)	1.19 (0.0–1.68)	1.19 (0.0–1.56)	0.4277	1.19 (0.0–1.69)	1.08 (0.0–1.49)	0.1499
Inhaled corticosteroid (n,%)	242 (21.1)	43 (20.9)	0.9514	242 (21.1)	43 (20.8)	0.9205
Systemic corticosteroid (n,%)	202 (25.8)	31 (20.8)	0.1998	203 (25.9)	30 (20.0)	0.1245
HFO (n,%)	293 (30.6)	52 (29.6)	0.7830	294 (30.7)	51 (28.8)	0.6124
Length of Invasive ventilation (day)	3 (1–19)	3 (1–16)	0.1965	3 (1–18)	3 (1–17)	0.3497
Length of Noninvasive ventilation (day)	30 (11–43)	18 (3–38)	<0.0001	30 (11–44)	18 (3–36)	<0.0001
Age at discharge (day)	78 (57–111)	71 (50–107)	0.0179	78 (57–111)	72 (50–107)	0.0281
Severe BPD (n,%)	127 (10.9)	22 (9.4)	0.4851	124 (10.7)	25 (10.6)	0.9784

Values are presented as median (first to third quartile) or numbers (%)

Cases were removed from the training dataset for CART analysis because the selected covariates had missing values.

BPD (bronchopulmonary dysplasia); SDS (standard deviation score); CAM (chorioamnionitis); PROM (premature rupture of membrane); RDS (respiratory distress syndrome). PDA means patent ductus arteriosus. IVH means intraventricular hemorrhage. RS score respiratory severity score; HFO, high-frequency oscillation

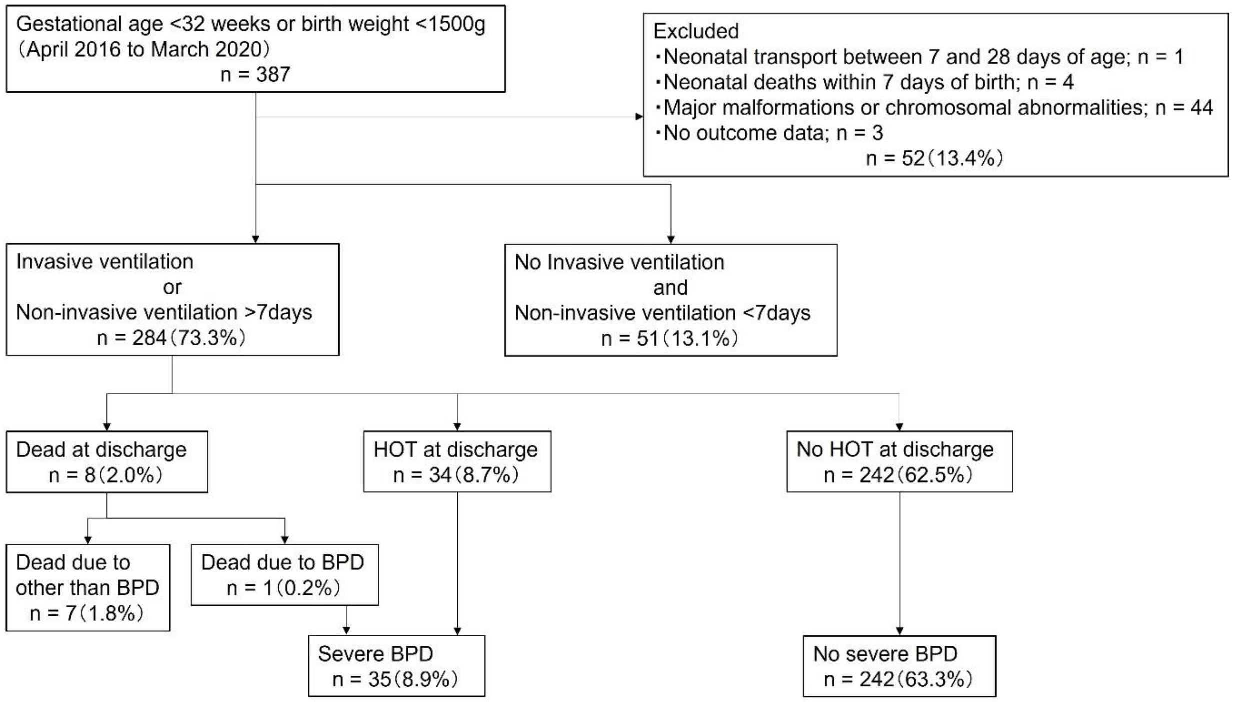

Supplement 3. Flowchart of the study population used in the validation dataset.

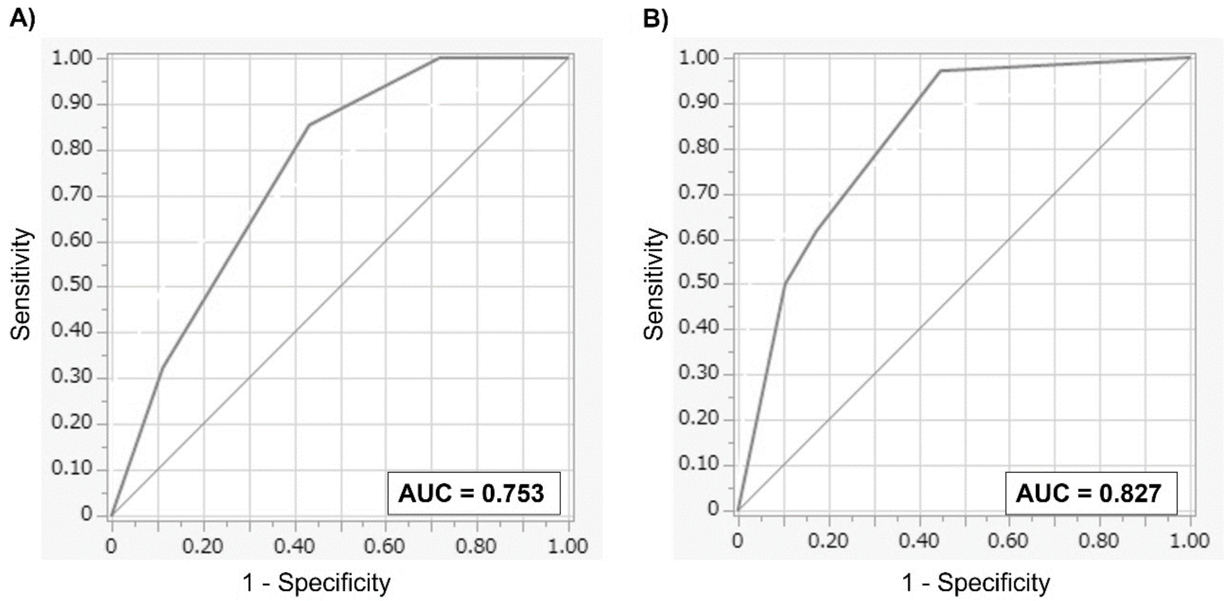

Supplement 4. Receiver operating characteristic (ROC) curves for the CART model for predicting severe BPD at 7 and 14 days of age (Validation dataset).

A) Postnatal day 7 model. B) Postnatal day 14 model.

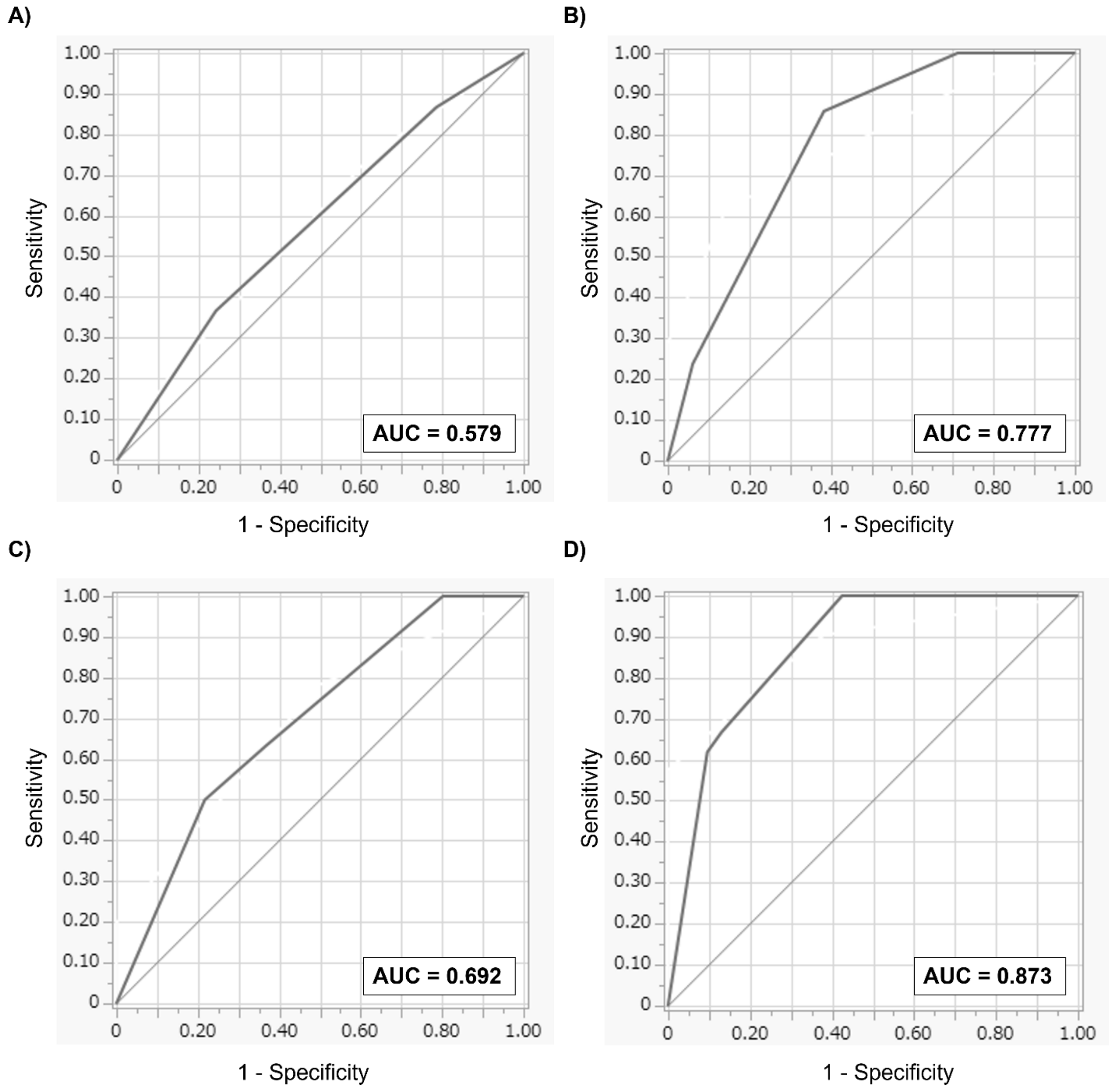

Supplement 5. Receiver operating characteristic (ROC) curves for the CART model for predicting severe BPD at 7 and 14 days of age (Subgroup of the validation data set).

A) Postnatal day 7 model (less than 28 weeks gestation). B) Postnatal day 7 model (maternal steroid administration). C) Postnatal day 14 model (less than 28 weeks gestation). D) Postnatal day 14 model (maternal steroid administration).

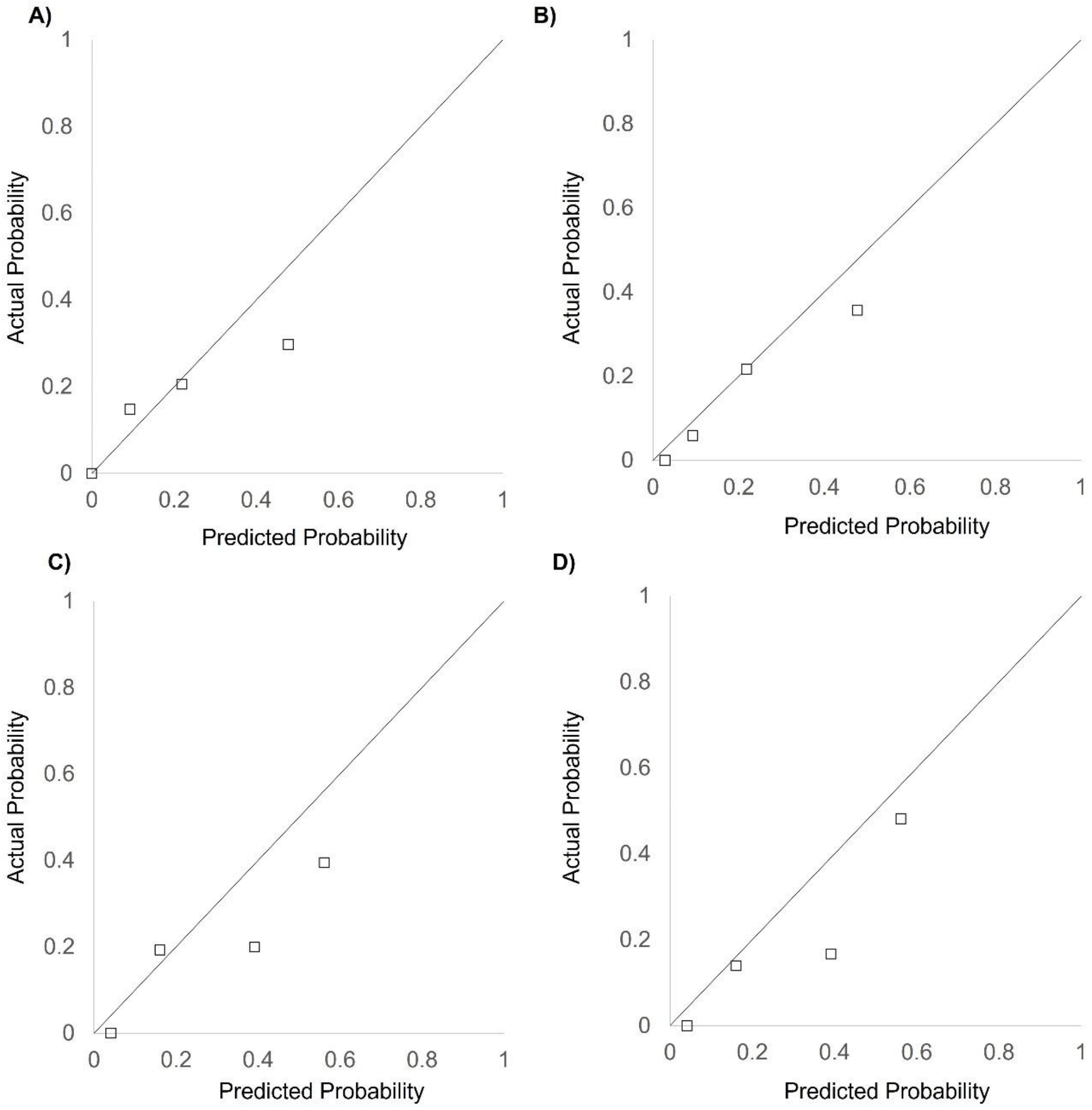

Supplement 6. Calibration plot using subgroup of the validation data set

A) Postnatal day 7 model (less than 28 weeks gestation). B) Postnatal day 7 model (maternal steroid administration). C) Postnatal day 14 model (less than 28 weeks gestation). D) Postnatal day 14 model (maternal steroid administration).
